# Heart Transplantation Requiring Permanent Pacemaker: Risk Factors and Outcomes

**DOI:** 10.3390/jcm15134895

**Published:** 2026-06-24

**Authors:** Michael Keller, Ye In Christopher Kwon, Yashar Haghighi, Vigneshwar Kasirajan, Zubair Hashmi

**Affiliations:** Division of Cardiothoracic Surgery, Department of Surgery, Pauley Heart Center, Virginia Commonwealth University School of Medicine, Richmond, VA 23298, USA; kellerms@vcu.edu (M.K.);

**Keywords:** pacemaker, heart transplantation, donation after cardiac death

## Abstract

**Background/Objectives**: Following heart transplantation (HT), a subset of patients will require an early or late permanent pacemaker (PPM). We explored risk factors and outcomes associated with PPM implantation in this population. **Methods**: Using the United Network for Organ Sharing (UNOS) database, we identified all adult patients undergoing HT from 2013 to 2023 who received a PPM early (prior to discharge) or late (>6 months post transplant). Propensity score matching (PSM) was used for control cohorts was. Primary outcomes included recipient survival at 30 days and 1 and 5 years. Predictors of early and late PPM, as well as post-PPM mortality, were assessed using Cox and logistic regression models. Kaplan–Meier survival curves were compared using a log-rank test. **Results**: Following PSM, the early PPM cohort included 354 patients, and the late PPM cohort included 554 patients. Early PPM patients showed similar 30-day and 1- and 5-year survival (*p* = 0.582, 0.421, and 0.2844 respectively) but lower rates of graft failure (1.1% vs. 4%, *p* = 0.017) and primary graft dysfunction (PGD) (1.7% vs. 4.2%, *p* = 0.046). Late PPM patients had reduced survival at 30 days and 1 year but not at 5 years (*p* < 0.001, *p* = 0.0023, 0.050 respectively). Neither early nor late PPM was independently associated with increased risk of mortality after HT. Donation after Circulatory Death (DCD) organs were associated with a lower risk of early PPM (aOR = 0.409, *p* = 0.020). Late PPM patients showed higher rates of PGD (2.5% vs. 0.5%, *p* = 0.007). **Conclusions**: Early or late PPM is not an independent risk factor for mortality after HT, but differing short-term morbidity and mortality are observed.

## 1. Introduction

Rhythm disturbances following heart transplantation (HT) are a well-known early complication; however, a subset of patients do not regain normal physiological conduction [1]. Prior studies have reported that anywhere from 4–20% of patients undergoing HT will require permanent pacemaker (PPM) implantation for ongoing sinus node dysfunction (SND) or atrioventricular (AV) blocks [2,3,4]. However, in recent years, the need for PPM implantation appears to have decreased, with rates reported to be as low as 5.8% [5,6]. These patients are more likely to be older, receive older donor hearts, and have lower ratios of reperfusion to aortic cross-clamp time and reperfusion to ischemia time [7]. The most predictive factor is surgical technique, with bicaval anastomoses protecting against the need for PPM implantation by decreasing anatomic disturbances [8]. Accordingly, bicaval anastomoses are associated with lower PPM rates (2.5% of bicaval patients vs. 5.7% of biatrial patients) [8].

Research examining the timing of pacemaker implantation reports differing outcomes between pacemakers implanted within the first 30 days post HT (early PPM) and those implanted after 30 days (late PPM) [5,9]. Conflicting data exist with respect to morbidity and mortality following PPM implantation, with some studies reporting significantly decreased five-year mortality while others report insignificant differences [4,5]. Recent reports also suggest an increased risk of graft failure in pacing-dependent hearts and a higher percentage of deaths attributed to infection [6,10].

Despite this, large cohort analyses in recent years remain limited [11]. A recent analysis of the UNOS database focusing on the impact of the 2018 organ allocation change found that PPM implantation has declined over time and is not associated with a survival benefit, but surgical technique was not reported [12]. Furthermore, no significant differences in pacemaker requirements have been observed between donation after circulatory death (DCD) and donation after brain death (DBD) recipients, although this may differ in patients suffering from primary graft dysfunction (PGD) [13,14]. Finally, survival outcomes for patients supported with early pacemakers suggested increased one-year mortality and graft failure in a single-center study, whereas another study showed increased five-year survival among late pacemaker recipients [3,6].

This study examines contemporary indications and outcomes of PPM implantation following heart transplantation in a propensity-matched cohort. We further explore risk factors and outcomes for patients receiving early and late pacemaker implantation and include an analysis of different procurement methods.

## 2. Materials and Methods

### 2.1. Study Design

Using the United Network for Organ Sharing (UNOS) Standard Analysis and Research file, we identified all adult patients (≥18 years) undergoing first-time HT from 1 January 2013 to 1 January 2023 who had at least 3 years of follow-up data. Patients were excluded if they underwent multi-organ transplantation or re-transplantation; we also excluded recipients with missing waitlist data and donors with missing procurement information. Patients were stratified by early or late pacemaker implantation. Early pacemaker patients were identified as those patients in the UNOS database who met the “events prior to discharge, permanent pacemaker” criterion. Late pacemaker patients were those coded as “having a pacemaker inserted since last follow-up”, with this having occurred more than 6 months post transplant (Figure S1). Accordingly, these groups were mutually exclusive. Patients with missing timing data were excluded. Subgroup analyses were performed, comparing DCD vs. DBD recipients. This study utilized de-identified data from the UNOS registry. As no protected health information or patient identifiers were included, this study was determined to be exempt from Institutional Review Board review at Virginia Commonwealth University, in accordance with 45 CFR 46.104(d)(4).

### 2.2. Outcomes

Primary outcomes included recipient survival rates at 30 days and 1 and 5 years. As the exact timing of pacemaker implantation was not available in the dataset, a surrogate implantation date was assigned. For patients who received an early pacemaker, survival was measured from the index date of transplantation. In the late pacemaker group, the index date was defined as the first follow-up assessment that documented permanent pacemaker implantation. Other outcomes included the incidence of primary graft dysfunction (PGD), length of hospital stay, post-operative stroke, dialysis, reintubation, airway dehiscence, acute rejection, and treatment for rejection within one year of transplantation.

### 2.3. Statistical Analyses

Propensity score matching (PSM) was performed using 1:1 nearest-neighbor matching without replacement, with a caliper of 0.2 standard deviations of the logit. Balance was defined as an absolute standardized mean difference <0.15 across covariates. Propensity scores were estimated using multivariable logistic regression incorporating previously identified baseline recipient and donor characteristics. Matched groups were compared using paired t-tests and McNemar’s test as appropriate. Donor and recipient characteristics were reported as percentages for categorical variables and as means ± SD for continuous variables. Comparisons of categorical variables were performed using the χ^2^ test or Fisher’s exact test, and continuous variables were compared using the Kruskal–Wallis rank-sum test. Risk of pacemaker implantation was first modeled with a univariate logistic hazards model; mortality was measured with a univariate Cox proportional hazards model. Variables demonstrating a significance of *p* < 0.10 in univariate analyses were included in the multivariate logistic and Cox regression analyses to evaluate risk of pacemaker implantation and the effect of pacemaker implantation on mortality. Adjusted odds ratios (aORs) and hazard ratios (HRs), along with 95% confidence intervals (CI), are reported for logistic and Cox regression models respectively. Kaplan–Meier curves were used to analyze mortality and were compared using the log-rank test. Analyses were conducted using SAS version 9.4 (SAS Inc., Cary, NC, USA), with two-sided *p*-values < 0.05 considered statistically significant. As no protected health information or patient identifiers were included in the dataset, this study was determined to be exempt from Institutional Review Board review at Virginia Commonwealth University, in accordance with 45 CFR 46.104(d)(4). 2.

## 3. Results

### 3.1. Early PPM

Among 909 pacemaker recipients, 354 patients (39%) received a PPM during the index hospitalization, while 29,050 patients did not receive an early PPM. Following PSM, 354 patients were included in both the early PPM and no-early PPM cohorts. Baseline recipient and donor characteristics before and after propensity matching are shown in Table 1. Matched cohorts demonstrated balance across covariates, as seen in Figure S2.

**Figure 1:**
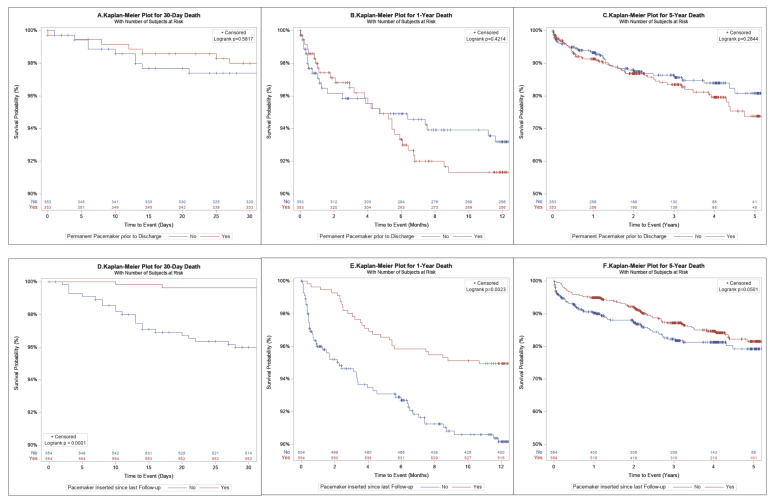
Kaplan–Meier survival analysis comparing no pacemaker implantation with early (**A**–**C**) and late (**D**–**F**) pacemaker implantation.

Patients with an early PPM experienced similar 30-day, 1-year, and 5-year mortality compared to matched patients who did not receive a pacemaker (*p* = 0.613, 0.378, 0.188; Table 2). Accordingly, survival did not differ (*p* = 0.582, 0.421, 0.2844; Figure 1). Graft failure within 1 year was lower for early PPM recipients (1.1% vs. 4%, *p* = 0.017) with lower rates of PGD (1.7% vs. 4.2%, *p* = 0.046; Table 2). Early PPM patients also had shorter lengths of hospital stay (25 days vs. 32 days, *p* = 0.001) and lower rates of post-operative dialysis (16.7% vs. 24.6%, *p* = 0.009; Table 2). Multivariable regression modeling revealed that use of DCD donor hearts was associated with a reduced risk of early PPM (aOR 0.409, *p* = 0.020), whereas donor perfusion was associated with increased risk of early PPM (aOR 4.053, *p* = 0.003; Table 3). Among patients who received an early PPM, longer ischemic time (HR 1.258, *p* = 0.002) and donor diabetes (HR 2.379, *p* = 0.012) were associated with an increased risk of mortality. Conversely, greater distance from the donor hospital to transplant center was associated with a lower risk of mortality (HR 0.999, *p* = 0.024; Table 4).

**Figure 2:**
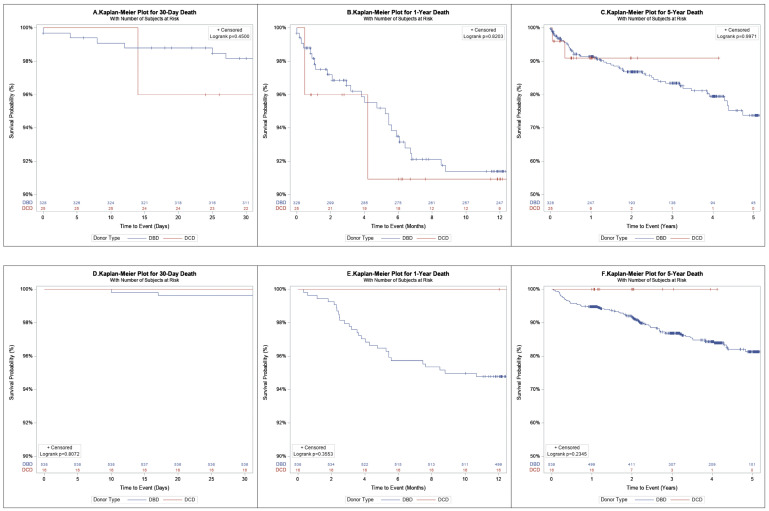
Kaplan–Meier survival analysis comparing DCD and DBD HT recipients who received early (**A**–**C**) or late (**D**–**F**) pacemakers.

When stratified by donor type (DCD vs. DBD), there were no differences in survival among early PPM recipients at 30 days (*p* = 0.450), 1 year (*p* = 0.820), or 5 years (*p* = 0.997; Figure 2). DCD patients with an early PPM had higher rates of PGD (24% vs. 2.7%, *p* < 0.001), left ventricular PGD (16% vs. 1.5%; *p* < 0.001), and right ventricular PGD (20% vs. 1.5%; *p* < 0.001; Table 4). Additionally, rates of post-operative dialysis were higher in DCD patients with early PPM (44% vs. 23.1%, *p* = 0.019; Table 4).

### 3.2. Late PPM

Among 909 pacemaker recipients, 555 patients (61%) received a pacemaker more than six months after transplantation. A total of 28,849 patients did not receive a late PPM. After PSM, the late PPM cohort included 554 patients, and the no-late PPM cohort included 554 patients. Baseline characteristics before and after PSM are shown in Table 1. Matched cohorts demonstrated balance across covariates, as seen in Figure S3.

Patients with a late PPM experienced higher rates of mortality at 30 days (4% vs. 0.4%, *p* < 0.001) and 1 year (9.2% vs. 5.1%, *p* = 0.007), whereas 5-year mortality was not significantly different (14.4% vs. 13.2%, *p* = 0.542; Table 2). Survival differed between the late PPM and no-late PPM cohorts at 30 days (*p* < 0.001) and 1 year (*p* = 0.002) but did not differ at 5 years (*p* = 0.050; Figure 1). There was no difference in graft failure (2.5% vs. 4%, *p* = 0.175), but PGD (2.5% vs. 0.5%, *p* = 0.007) and right ventricular PGD (2.4% vs. 0.5%, *p* = 0.018) were higher in the late PPM cohort. Additionally, the rate of post-operative stroke was higher in the late PPM cohort (5.8% vs. 2.7%, *p* = 0.011; Table 2).

None of the variables included in the logistic regression were associated with an increased risk of late PPM implantation (Table 3). In adjusted mortality analysis, PPM insertion since last follow-up was not independently associated with higher mortality risk (HR 0.748, *p* = 0.077; Table 5). However, increased recipient age (HR 1.015, *p* = 0.043), longer ischemic time (HR 1.187, *p* = 0.039), and prior recipient cardiac surgery (HR 1.397, *p* = 0.048) were associated with higher mortality among late PPM recipients (Table 5).

Stratification by DCD and DBD organ transplantation did not reveal differences in mortality or survival rates at 30 days, 1 year, or 5 years or other outcomes (Table 4, Figure 2).

## 4. Discussion

A propensity score-matched analysis of the UNOS database to identify indications and outcomes for permanent pacemakers following heart transplantation yielded several important findings for future consideration. Early PPM was not associated with an increased risk of mortality at 30 days, 1 year, or 5 years. These patients also had lower rates of primary graft dysfunction and post-operative dialysis and shorter hospital stays when compared to matched patients with no pacemaker. This could be due to a myriad of reasons likely related to clinical considerations rather than a protective factor of the pacemaker. Donors to early PPM recipients were more likely to be older and have a higher body mass index (BMI). In an adjusted model, DCD donors were associated with a lower risk of early PPM; however, early pacemaker patients who received DCD organs had higher rates of PGD, LVPGD, and post-operative dialysis. Late PPM recipients experienced higher 30-day and 1-year mortality; however, this increase was not seen at 5 years. These late PPM recipients are also associated with higher rates of PGD and RVPGD. Despite observed increases in mortality, in adjusted models, late PPM was not independently associated with increased mortality; however, older recipient age, longer ischemic time, and prior cardiac surgeries were associated with increased risk of mortality for late PPM recipients.

We identified 3.1% of our population who required PPM placement, consistent with recent reports [3,12,15]. Our cohort, as well as those studied by Rivinius et al. and Roest et al., demonstrated higher rates of late PPM placement, whereas Mallidi et al. reported increased rates of early PPM placement [6,15,16]. Interpretation of these differences should consider the era of transplantation, as Mallidi’s study included an earlier time period [15]. A recent analysis of the UNOS database by Doulamis et al. reported PPM incidence and risk factors, including decreased risk in later years [12]. Our study builds upon their prior work by including stratifications into early and late PPM requirement and including commentary on DCD and DBD organ recipients.

Herrmann and Luebbert each reported that early pacemaker implantation was more commonly indicated for transplant recipients who suffered from SND [7,17]. These patients exhibited a trend toward being male and having longer ischemia times than patients who did not need a PPM after SND; however, these trends were not significant [7]. It has been reported that the most significant impact on the need for PPM in SND patients is exerted by the biatrial operative technique due to surgical manipulation of the intrinsic conduction system [4,6]. In our cohort, early PPM recipients were also more likely to be male; however, they had a shorter ischemic time. In accordance with other reports, PPM patients were more likely to have received a donor organ from an older patient, but donor age was not independently associated with increased risk of early PPM [16,18]. Recipients of organs from donors with a higher BMI, hypertension, or diabetes were more likely to require PPM, but these factors were not independently associated with PPM placement in multivariable regression models. In the non-transplant population, diabetes has been shown to be an independent risk factor for PPM need, with the proposed physiological changes for PPM need likely still present in transplanted organs.

Short- and long-term mortality rates for early PPM after HT are similar to those in HT patients without PPM both in our study and in those done by Roest and Jones but differ from the results reported in a study completed by Rivinius [6,16,18]. We further report that early PPM is not an independent risk factor for mortality following HT. Despite comparable survival, early PPM recipients were associated with lower rates of primary graft dysfunction and reduced need for post-operative dialysis but experienced higher rates of graft failure within one year.

In adjusted analysis, we report that mortality for early PPM patients is increased by donor diabetes and prolonged ischemic time. Diabetes is a well-known risk factor for pacemaker implantation, with proposed mechanisms including diabetic neuropathy, cardiac fibrosis, and diabetic cardiomyopathy. These can lead to impaired electrical conduction, increased oxidative stress and chronic inflammation leading to structural remodeling and potential alterations in electrical conduction [19,20,21]. While neuropathy and fibrosis are unlikely to directly affect transplanted hearts, microangiopathic injury and intrinsic abnormalities in electrical conduction may persist following transplantation. Increased ischemic time has been shown to be a predictor for both mortality and other complications [22,23]. Our data continue to show increased ischemic time as an independent risk factor for mortality. Despite this, we also show that increased distance from the donor hospital to the transplant center is associated with decreased mortality. Although counterintuitive, center practices, selection bias, transport type, and preservation technologies may help to explain this. Recent studies have not shown distance to influence short- or long-term survival, although in the population receiving an early PPM, there is a clear impact on mortality [24].

When analyzing early PPM recipients stratified by donation type, a distinct pattern emerged among DCD recipients. In our propensity-matched cohort, DCD was associated with a reduced risk of PPM and did not affect mortality. However, early PPM patients receiving DCD organs had significantly higher rates of post-operative dialysis and higher rates of PGD. PGD remains a nebulous diagnosis with several suggested mechanisms, including hypothermic ischemia, warm ischemic time, ischemia–reperfusion injury, and vasoplegic recipient responses [25]. Consistent with other studies, our results propose that DCD donors may experience increased morbidity in the peri-operative period, but this does not portend toward worsened long-term outcomes [14,26,27].

The reported incidence of late PPM following heart transplantation varies widely, largely due to differences in definitions and patient populations. We report an incidence similar to that reported by Rivinius and Noworolski but higher than rates reported in several other studies [6,16,18,28]. Late PPM implantation is most commonly indicated for symptomatic AV blocks [6,18]. Consistent with early PPM, the most significant risk factor is that of the operative technique, with the biatrial technique increasing risk of late PPM [15,28]. A few other risk factors have been identified in the literature, mainly regarding allograft vasculopathy or in concordance with acute rejection episodes [15,16]. Before propensity matching, members of our late PPM cohort experienced shorter ischemic times, were more likely to be supported by mechanical circulatory support prior to transplantation, and were more likely to have received an automatic implantable cardiac defibrillator or undergone other prior cardiac surgeries. We also report that late PPM recipients were more likely to receive older donor hearts and organs from donors with a higher BMI, as has been reported in prior studies [16]. However, in adjusted analyses, neither donor age nor donor BMI was independently associated with an increased risk of late PPM.

Like incidence, survival and mortality rates following late PPM are discordant amongst the literature. Depending on the study, late PPM has been associated with improved 1- and 5-year survival compared with no PPM; no differences in survival at 1, 5, and 10 years compared with no PPM and early PPM; and independently decreased survival in adjusted analyses [6,16,18]. These differences may be attributed to geographic variations, sample size, or the era of transplantation across prior studies [6,16,18,28]. Our study of 555 patients in the United States revealed increased mortality at 30 days and 1 year, while 5-year mortality did not differ between patients with a late pacemaker and those without. Interestingly, late PPM was associated with increases in both PGD and RVPGD, suggesting a correlation between early graft dysfunction and later conduction issues. This may be due to ischemia–reperfusion injury causing microangiopathic damage to the sinus and atrioventricular nodal arteries, which contain limited collateral supply. Resultant microvascular insufficiency and fibrosis of the conduction system may manifest initially as PGD and later lead to a conduction block requiring late PPM implantation. Right ventricular involvement in PGD is particularly relevant, given the right coronary artery’s dual role in perfusing both the right ventricle and the AV node in most patients. Despite worse short-term outcomes, late PPM was not independently associated with mortality in heart transplant recipients. Like all heart transplant recipients, increased recipient age was identified as a risk factor for increased mortality in late PPM patients [29]. Prior cardiac surgery was strongly associated with increased risk for mortality following late PPM. While distance from the donor hospital to the transplant center was not an independent risk factor for mortality, increased ischemic time was a risk factor. This is consistent with the mechanism discussed earlier: ischemia–reperfusion injury promoting microvascular changes and nodal fibrosis may predispose the transplanted heart to late conduction disease. These findings suggest that early graft dysfunction and a late pacemaker requirement may be consequences of ischemic injury to the conduction system rather than independent events.

Several findings bear directly on clinical decision-making. First, the fact that neither early nor late PPM was independently associated with mortality reframes the device as more a marker of recipient and graft trajectory than a driver of poor outcome and supports the counseling of patients and families that pacemaker dependency after transplantation does not, by itself, portend worse survival. Second, the divergent profiles of early and late PPM are prognostically useful: early PPM recipients had lower rates of graft failure, primary graft dysfunction, and dialysis (with an expectedly longer index admission reflecting in-hospital device placement), whereas late PPM was associated with higher rates of early mortality, PGD, and stroke, without a difference in mortality at five years. This suggests that the late emergence of conduction disease may flag a higher-risk trajectory warranting closer surveillance rather than an isolated electrical event. Third, as DCD utilization expands, the lower early PPM risk observed in DCD recipients, alongside their higher rates of PGD and dialysis, helps calibrate postoperative expectations as programs scale DCD. Finally, the association between donor perfusion and increased early PPM risk, while hypothesis-generating, warrants prospective attention as machine perfusion is more widely adopted and is a reason to maintain heightened early rhythm monitoring in perfused allografts. We acknowledge that several identified risk factors, including donor age, ischemic time, and recipient comorbidity, are not directly modifiable through allocation; their value lies in expectation setting, in identifying recipients who merit earlier electrophysiologic evaluation, and in tempering the assumption that PPM independently worsens transplant survival.

The limitations of this study primarily stem from its retrospective design and reliance on a national registry. Granularity regarding biatrial versus bicaval operative techniques is no longer reported in the UNOS database, despite being found, in many studies, to affect pacemaker implantation. Because bicaval anastomosis reduces conduction-system disturbance and is associated with lower PPM rates, the inability to adjust for technique is an important limitation. The predominance of bicaval anastomosis in the contemporary US era may attenuate but does not eliminate this concern, and center-level studies with operative detail are needed to assess the contribution of technique directly. Additionally, granular timing and indications for pacemaker implantation could not be determined. This is of particular interest for rejection episodes and graft failure that may occur at any point after transplantation and affect late PPM. Additionally, this may impact survival analyses, as they relied on surrogate index dates. Institutional and surgeon-level variation in the management of post-operative bradyarrhythmias, including the duration of epicardial pacing, may also have influenced the observed outcomes. Finally, because DCD transplantation is only performed at select centers, center-specific practices and donor–recipient selection may further impact these findings.

## 5. Conclusions

For patients who receive pacemakers after heart transplantation, the timeframe of implantation and indications for pacemakers play a role in survival and outcomes. Risk factors and etiologies differed between early and late PPM recipients. Risk of early PPM was decreased by the use of DCD donors, but early PPM recipients with DCD donors were more likely to have suffered PGD and LVPGD. Mortality in early PPM recipients was increased by prolonged ischemic time and donor diabetes, while mortality in late PPM recipients was increased by recipient age, ischemic time, and prior cardiac surgeries. Most importantly, we found that early PPM implantation is not a risk factor for mortality and that, while survival is similar, these patients are associated with decreased lengths of hospital stay, rates of graft failure, PGD, and post-operative dialysis. Although late PPM was not independently associated with mortality, these patients experienced higher mortality at 30 days and 1 year following transplantation.

## Figures and Tables

**Table 1:** Baseline characteristics for early and late PPM before and after propensity score matching.

**Variables**	**Before PSM**				**After PSM**			
	**Total**	**Early PPM (N = 354)**	**No Early PPM (N = 29,050)**	***p Value***	**Total**	**Early PPM (N = 354)**	**No Early PPM (N = 354)**	***p Value***
**Recipient**								
**Age, MEAN (Standard Deviation, STD)**	55.52 (12.45)	55.22 (11.89)	55.53 (12.45)	*0.6433*	55.48 (12.02)	55.22 (11.89)	55.74 (12.17)	*0.5634*
**Sex, N (%)**				***0.0035***				*0.3554*
Male	20,777	275 (77.7%)	20,502 (70.6%)		560	275 (77.7%)	285 (80.5%)	
Female	8627	79 (22.3%)	8548 (29.4%)		148	79 (22.3%)	69 (19.5%)	
**Body Mass Index, MEAN (STD)**	24.52 (6.26)	28.24 (5.09)	25.08 (4.53)	*0.7955*	28.28 (6.03)	28.77 (7.05)	27.79 (4.78)	*0.3021*
**Race, N (%)**				***0.0349***				*0.6028*
Non-Hispanic/Latino White	18,389	196 (55.4%)	18,193 (62.6%)		387	196 (55.4%)	191 (54%)	
Non-Hispanic/Latino Black	6094	96 (27.1%)	5998 (20.7%)		209	96 (27.1%)	113 (31.9%)	
Hispanic/Latino	3364	41 (11.6%)	3323 (11.4%)		74	41 (11.6%)	33 (9.3%)	
Non-Hispanic/Latino Asian	1077	15 (4.2%)	1062 (3.7%)		27	15 (4.2%)	12 (3.4%)	
Non-Hispanic/Latino Other	480	6 (1.7%)	474 (1.6%)		11	6 (1.7%)	5 (1.4%)	
**Distance from Donor Hospital to Transplant Center, MEAN (STD)**	300.04 (280.63)	308.4 (276.25)	299.94 (280.68)	*0.5731*	304.06 (273.17)	308.4 (276.25)	299.71 (270.37)	*0.6726*
**Ischemic Time in Hours, MEAN (STD)**	4.57 (2.43)	3.75 (1.35)	4.58 (2.44)	***<0.0001***	3.75 (1.46)	3.76 (1.35)	3.74 (1.57)	*0.8325*
**Preop Hemodynamics, MEAN (STD)**								
Mean Pulmonary Artery Pressure	27.74 (11.01)	27.5 (11.39)	27.74 (11.01)	*0.6931*	27.74 (11.32)	27.51 (11.14)	27.98 (11.52)	*0.5837*
Pulmonary Capillary Wedge Pressure	15.9 (8.87)	17.05 (8.53)	15.89 (8.87)	***0.0174***	17.33 (8.58)	16.97 (8.23)	17.68 (8.91)	*0.2736*
Cardiac Output	4.76 (1.58)	4.57 (1.48)	4.77 (1.58)	***0.0214***	4.59 (1.46)	4.58 (1.44)	4.6 (1.47)	*0.8475*
**Cardiac Support Devices at Transplant, N (%)**								
Extra-Corporeal Membrane Oxygenation	2085	21 (5.9%)	2064 (7.1%)	*0.3928*	39	21 (5.9%)	18 (5.1%)	*0.6212*
Intra-Aortic Balloon Pump	5121	78 (22%)	5043 (17.4%)	***0.0212***	161	78 (22%)	83 (23.5%)	*0.6539*
Ventilator	922	10 (2.8%)	912 (3.1%)	*0.7357*	19	10 (2.8%)	9 (2.5%)	*0.8161*
**Comorbidities, N (%)**								
Steroid	4203	32 (9%)	4171 (14.4%)	***0.0045***	65	32 (9%)	33 (9.3%)	*0.8964*
Smoking	13,386	140 (39.6%)	13,246 (45.6%)	***0.0231***	284	140 (39.6%)	144 (40.7%)	*0.7591*
Malignancy	2888	20 (5.7%)	2868 (9.9%)	***0.0080***	34	20 (5.7%)	14 (4%)	*0.2916*
Automatic Implantable Cardioverter Defibrillator	13,542	255 (72%)	13,287 (45.7%)	***<0.0001***	519	255 (72%)	264 (74.6%)	*0.4445*
Prior Cardiac Surgery	7463	135 (38.1%)	7328 (25.2%)	***<0.0001***	266	135 (38.1%)	131 (37%)	*0.7563*
**Donor**								
**Age, MEAN (STD)**	32.24 (10.36)	35.05 (10.89)	32.2 (10.35)	***<0.0001***	34.88 (10.52)	35.05 (10.89)	34.7 (10.15)	*0.6607*
**Sex, N (%)**				*0.4628*				*0.8001*
Male	20,744	256 (72.3%)	20,488 (70.5%)		515	256 (72.3%)	259 (73.2%)	
Female	8660	98 (27.7%)	8562 (29.5%)		193	98 (27.7%)	95 (26.8%)	
**Body Mass Index, Mean (STD)**	27.58 (6.17)	28.77 (6.67)	27.57 (6.16)	***0.0003***	28.99 (6.67)	28.77 (6.67)	29.21 (6.68)	*0.3814*
**Race, N (%)**				*0.0786*				*0.4043*
Non-Hispanic/Latino White	18,079	214 (60.5%)	17,865 (61.5%)		453	214 (60.5%)	239 (67.5%)	
Non-Hispanic/Latino Black	4844	45 (12.7%)	4799 (16.5%)		81	45 (12.7%)	36 (10.2%)	
Hispanic/Latino	5475	83 (23.5%)	5392 (18.6%)		151	83 (23.5%)	68 (19.2%)	
Non-Hispanic/Latino Asian	545	8 (2.3%)	537 (1.9%)		16	8 (2.3%)	8 (2.3%)	
Non-Hispanic/Latino Other	461	4 (1.1%)	457 (1.6%)		7	4 (1.1%)	3 (0.9%)	
**Left Ventricular Ejection Fraction, MEAN (STD)**	61.77 (6.72)	62.26 (6.85)	61.77 (6.72)	*0.1738*	62.06 (6.56)	62.26 (6.84)	61.87 (6.26)	*0.4382*
**Comorbidities, N (%)**								
Diabetes	1175	24 (6.8%)	1151 (4%)	***0.0071***	46	24 (6.8%)	22 (6.2%)	*0.7604*
Smoking	2762	32 (9%)	2730 (9.4%)	*0.8185*	73	32 (9%)	41 (11.6%)	*0.2660*
Cocaine	5976	74 (20.9%)	5902 (20.3%)	*0.7849*	161	74 (20.9%)	87 (24.6%)	*0.2438*
Alcohol abuse	5623	61 (17.2%)	5562 (19.2%)	*0.3626*	131	61 (17.2%)	70 (19.8%)	*0.3837*
Malignancy	358	7 (2%)	351 (1.2%)	*0.1897*	12	7 (2%)	5 (1.4%)	*0.5604*
Hypertension	4352	82 (23.2%)	4270 (14.7%)	***<0.0001***	158	82 (23.2%)	76 (21.5%)	*0.5881*
Intravenous drug use	4614	52 (14.7%)	4562 (15.7%)	*0.6019*	104	52 (14.7%)	52 (14.7%)	*1.0000*
Myocardial Infarction	288	3 (0.9%)	285 (1%)	*0.7997*	5	3 (0.9%)	2 (0.6%)	*0.6536*
**Donor Type, N (%)**				*0.7534*				*0.4054*
Donation after Brain Death	27,449	329 (92.9%)	27,120 (93.4%)		670	329 (92.9%)	341 (96.3%)	
Donation after Cardiac Death	1955	25 (7.1%)	1930 (6.6%)		38	25 (7.1%)	13 (3.7%)	
**Donor Perfusion, N (%)**	2518	25 (7.1%)	2493 (8.6%)	*0.3098*	56	25 (7.1%)	31 (8.8%)	*0.4034*
**Variables**	**Before PSM**				**After PSM**			
	**Total**	**Late PPM (N = 555)**	**No Late PPM (N = 28,849)**	***p Value***	**Total**	**Late PPM (N = 554)**	**No Late PPM (N = 554)**	***p Value***
**Recipient**								
**Age, MEAN (STD)**	55.52 (12.45)	54.61 (12.22)	55.54 (12.45)	*0.0807*	54.48 (12.18)	54.59 (12.23)	54.37 (12.14)	*0.7654*
**Sex, N (%)**				*0.1627*				*0.5449*
Male	20,777	407 (73.3%)	20,370 (70.6%)		803	406 (73.3%)	397 (71.7%)	
Female	8627	148 (26.7%)	8479 (29.4%)		305	148 (26.7%)	157 (28.3%)	
**Body Mass Index, Mean (STD)**	24.52 (6.26)	21.37 (6.79)	24.58 (5.98)	*0.9822*	49.53 (719.64)	71.44 (1017.7)	27.63 (5.4)	*0.3111*
**Race, N (%)**				***0.0005***				*0.8080*
Non-Hispanic/Latino White	18,389	299 (53.9%)	18,090 (62.7%)		607	298 (53.8%)	309 (55.8%)	
Non-Hispanic/Latino Black	6094	144 (26%)	5950 (20.6%)		293	144 (26%)	149 (26.9%)	
Hispanic/Latino	3364	79 (14.2%)	3285 (11.4%)		146	79 (14.3%)	67 (12.1%)	
Non-Hispanic/Latino Asian	1077	26 (4.7%)	1051 (3.6%)		48	26 (4.7%)	22 (4%)	
Non-Hispanic/Latino Other	480	7 (1.3%)	473 (1.6%)		14	7 (1.3%)	7 (1.3%)	
**Distance from Donor Hospital to Transplant Center, MEAN (STD)**	300.04 (280.63)	290.74 (272.56)	300.22 (280.78)	*0.4303*	285.56 (264.26)	289.61 (271.51)	281.5 (256.98)	*0.6094*
**Ischemic Time in Hours, MEAN (STD)**	4.57 (2.43)	3.75 (1.31)	4.59 (2.45)	***<0.0001***	3.7 (1.31)	3.75 (1.3)	3.64 (1.31)	*0.1524*
**Preop Hemodynamics, MEAN (STD)**								
Mean Pulmonary Artery Pressure	27.74 (11.01)	27.51 (11.08)	27.74 (11.01)	*0.6279*	27.16 (10.6)	27.52 (10.86)	26.79 (10.33)	*0.2505*
Pulmonary Capillary Wedge Pressure	15.9 (8.87)	17.8 (8.68)	15.86 (8.87)	***<0.0001***	17.64 (8.58)	17.65 (8.37)	17.63 (8.79)	*0.9566*
Cardiac Output	4.76 (1.58)	4.58 (1.48)	4.77 (1.58)	***0.0059***	4.58 (1.48)	4.58 (1.45)	4.58 (1.52)	*0.9947*
**Cardiac Support Devices at Transplant, N (%)**								
Extra-Corporeal Membrane Oxygenation	2085	27 (4.9%)	2058 (7.1%)	***0.0391***	58	27 (4.9%)	31 (5.6%)	*0.5895*
Intra-Aortic Balloon Pump	5121	130 (23.4%)	4991 (17.3%)	***0.0002***	260	130 (23.5%)	130 (23.5%)	*1.0000*
Ventilator	922	10 (1.8%)	912 (3.2%)	*0.0687*	21	10 (1.8%)	11 (2%)	*0.8256*
**Comorbidities, N (%)**								
Steroid	4203	37 (6.7%)	4166 (14.4%)	***<0.0001***	75	37 (6.7%)	38 (6.9%)	*0.9048*
Smoking	13,386	232 (41.8%)	13,154 (45.6%)	*0.0754*	464	232 (41.9%)	232 (41.9%)	*1.0000*
Malignancy	2888	37 (6.7%)	2851 (9.9%)	***0.0117***	83	37 (6.7%)	46 (8.3%)	*0.3044*
Automatic Implantable Cardioverter Defibrillator	13,542	411 (74.1%)	13,131 (45.5%)	***<0.0001***	824	410 (74%)	414 (74.7%)	*0.7831*
Prior Cardiac Surgery	7463	196 (35.3%)	7267 (25.2%)	***<0.0001***	391	195 (35.2%)	196 (35.4%)	*0.9499*
**Donor**								
**Age, MEAN (STD)**	32.24 (10.36)	33.59 (10.9)	32.21 (10.35)	***0.0019***	33.51 (10.76)	33.58 (10.9)	33.44 (10.62)	*0.8212*
**Sex, N (%)**				*0.3739*				*0.4264*
Male	20,744	401 (72.3%)	20,343 (70.5%)		788	400 (72.2%)	388 (70%)	
Female	8660	154 (27.8%)	8506 (29.5%)		320	154 (27.8%)	166 (30%)	
**Body Mass Index, Mean (STD)**	27.58 (6.17)	28.36 (6.23)	27.57 (6.17)	***0.0029***	28.38 (6.58)	28.32 (6.19)	28.43 (6.96)	*0.7860*
**Race, N (%)**				*0.0637*				*0.8744*
Non-Hispanic/Latino White	18,079	333 (60%)	17,746 (61.5%)		680	333 (60.1%)	347 (62.6%)	
Non-Hispanic/Latino Black	4844	77 (13.9%)	4767 (16.5%)		144	77 (13.9%)	67 (12.1%)	
Hispanic/Latino	5475	121 (21.8%)	5354 (18.6%)		237	120 (21.7%)	117 (21.1%)	
Non-Hispanic/Latino Asian	545	10 (1.8%)	535 (1.9%)		21	10 (1.8%)	11 (2%)	
Non-Hispanic/Latino Other	461	14 (2.5%)	447 (1.6%)		26	14 (2.5%)	12 (2.2%)	
**Left Ventricular Ejection Fraction, MEAN (STD)**	61.77 (6.72)	62.09 (6.68)	61.77 (6.72)	*0.2613*	62.14 (6.56)	62.1 (6.69)	62.18 (6.44)	*0.8401*
**Comorbidities, N (%)**								
Diabetes	1175	21 (3.8%)	1154 (4%)	*0.7966*	39	21 (3.8%)	18 (3.3%)	*0.6248*
Smoking	2762	62 (11.2%)	2700 (9.4%)	*0.1472*	123	62 (11.2%)	61 (11%)	*0.9238*
Cocaine	5976	154 (27.8%)	5822 (20.2%)	***<0.0001***	322	154 (27.8%)	168 (30.3%)	*0.3543*
Alcohol abuse	5623	109 (19.6%)	5514 (19.1%)	*0.7548*	231	109 (19.7%)	122 (22%)	*0.3363*
Malignancy	358	8 (1.4%)	350 (1.2%)	*0.6272*	15	8 (1.4%)	7 (1.3%)	*0.7949*
Hypertension	4352	117 (21.1%)	4235 (14.7%)	***<0.0001***	218	116 (20.9%)	102 (18.4%)	*0.2901*
Intravenous drug use	4614	81 (14.6%)	4533 (15.7%)	*0.4731*	171	81 (14.6%)	90 (16.3%)	*0.4542*
Myocardial Infarction	288	11 (2%)	277 (1%)	***0.0155***	18	10 (1.8%)	8 (1.4%)	*0.6346*
**Donor Type, N (%)**				***0.0003***				*0.0871*
Donation after Brain Death	27,449	539 (97.1%)	26,910 (93.3%)		1065	538 (97.1%)	527 (95.1%)	
Donation after Cardiac Death	1955	16 (2.9%)	1939 (6.7%)		43	16 (2.9%)	27 (4.9%)	
**Donor Perfusion, N (%)**	2518	29 (5.2%)	2489 (8.6%)	***0.0045***	57	28 (5.1%)	29 (5.2%)	*0.8918*

**Table 2:** Outcomes following pacemaker implantation.

Variables	Total	Early PPM (N = 354)	No Early PPM (N = 354)	*p Value*	Total	Late PPM (N = 554)	No Late PPM (N = 554)	*p Value*
**Death**								
Death overall, N (%)	98	42 (11.9%)	56 (15.8%)	*0.1276*	160	82 (14.8%)	78 (14.1%)	*0.7324*
Death within 30 days, N (%)	16	9 (2.5%)	7 (2%)	*0.6130*	24	22 (4%)	2 (0.4%)	***<0.0001***
Death within 1 year, N (%)	50	22 (6.2%)	28 (7.9%)	*0.3788*	79	51 (9.2%)	28 (5.1%)	***0.0072***
Death within 5 years, N (%)	96	42 (11.9%)	54 (15.3%)	*0.1877*	153	80 (14.4%)	73 (13.2%)	*0.5421*
**Graft Failure**								
Graft Failure overall, N (%)	29	11 (3.1%)	18 (5.1%)	*0.1844*	36	14 (2.5%)	22 (4%)	*0.1752*
Graft Failure within 30 days, N (%)	10	3 (0.9%)	7 (2%)	*0.2027*	6	5 (0.9%)	1 (0.2%)	*0.1015*
Graft Failure within 1 year, N (%)	18	4 (1.1%)	14 (4%)	***0.0170***	19	11 (2%)	8 (1.4%)	*0.4875*
Graft Failure within 5 years, N (%)	29	11 (3.1%)	18 (5.1%)	*0.1844*	36	14 (2.5%)	22 (4%)	*0.1752*
**Primary Outcome**								
Primary Graft Dysfunction, N (%)	21	6 (1.7%)	15 (4.2%)	***0.0462***	17	14 (2.5%)	3 (0.5%)	***0.0072***
Left Primary Graft Dysfunction, N (%)	13	4 (1.1%)	9 (2.5%)	*0.1616*	12	9 (1.6%)	3 (0.5%)	*0.0816*
Right Primary Graft Dysfunction, N (%)	13	3 (0.9%)	10 (2.8%)	*0.0501*	16	13 (2.4%)	3 (0.5%)	***0.0118***
**Secondary Outcome**								
Length of Hospital Stay, MEAN (STD)	28.42 (29.94)	32.04 (27.18)	24.87 (32.07)	***0.0016***	26.41 (26.07)	27.33 (23.55)	25.47 (28.38)	*0.2370*
Stroke, N (%)	30	13 (3.7%)	17 (4.8%)	*0.4555*	47	32 (5.8%)	15 (2.7%)	***0.0113***
Dialysis, N (%)	146	59 (16.7%)	87 (24.6%)	***0.0093***	199	92 (16.6%)	107 (19.3%)	*0.2404*
Acute Rejection, N (%)	67	26 (7.3%)	41 (11.6%)	*0.0541*	88	48 (8.7%)	40 (7.2%)	*0.3741*
Treated for Rejection within 1 year, N (%)	89	42 (11.9%)	47 (13.3%)	*0.5708*	141	61 (11%)	80 (14.4%)	*0.0868*
Reintubation, N (%)	20	8 (2.3%)	12 (3.4%)	*0.3642*	10	7 (1.3%)	3 (0.5%)	*0.2039*
Airway Dehiscence, N (%)	6	3 (0.9%)	3 (0.9%)	*0.9999*	3	1 (0.2%)	2 (0.4%)	*0.5632*

**Table 3:** Multivariate logistic regression for risk of early and late PPM.

Variable	Early PPM			Late PPM		
	aOR	[95% CI]	*p Value*	aOR	[95% CI]	*p Value*
**Recipient**						
**Age**	0.997	[0.984–1.011]	*0.6927*	1.003	[0.993–1.014]	*0.5447*
**Sex**						
Male	REF			REF		
Female	1.179	[0.752–1.850]	*0.4724*	0.977	[0.702–1.360]	*0.8919*
**Body Mass Index**	1.026	[0.997–1.055]	*0.0775*	1.025	[0.999–1.050]	*0.0561*
**Race**						
Non-Hispanic/Latino White	REF			REF		
Non-Hispanic/Latino Black	0.807	[0.561–1.160]	*0.2464*	1.004	[0.752–1.339]	*0.9806*
Hispanic/Latino	1.157	[0.681–1.966]	*0.5887*	1.320	[0.901–1.933]	*0.1536*
Non-Hispanic/Latino Asian	1.262	[0.552–2.882]	*0.5815*	1.316	[0.715–2.424]	*0.3781*
Non-Hispanic/Latino Other	1.287	[0.370–4.470]	*0.6914*	0.973	[0.329–2.877]	*0.9607*
**Distance from Donor Hospital to Transplant Center**	1.000	[0.999–1.001]	*0.4504*	1.000	[0.999–1.000]	*0.5508*
**Ischemic Time in Hours**	1.003	[0.881–1.141]	*0.9677*	1.090	[0.966–1.231]	*0.1626*
**Preop Hemodynamics**						
Mean Pulmonary Artery Pressure	1.121	[0.325–3.867]	*0.8566*	0.924	[0.347–2.460]	*0.8750*
Pulmonary Capillary Wedge Pressure	1.040	[0.463–2.336]	*0.9250*	0.817	[0.430–1.553]	*0.5369*
Cardiac Output	0.745	[0.215–2.577]	*0.6420*	1.140	[0.434–2.994]	*0.7901*
**Cardiac Support Devices at Transplant**						
Extra-Corporeal Membrane Oxygenation	1.323	[0.658–2.661]	*0.4325*	0.885	[0.491–1.595]	*0.6838*
Ventilator	1.005	[0.371–2.719]	*0.9924*	1.058	[0.413–2.709]	*0.9065*
**Comorbidities**						
Steroid	0.959	[0.549–1.676]	*0.8833*	0.943	[0.583–1.524]	*0.8101*
Smoking	0.951	[0.692–1.306]	*0.7548*	0.988	[0.769–1.270]	*0.9266*
Malignancy	1.186	[0.568–2.475]	*0.6501*	0.803	[0.505–1.276]	*0.3530*
Prior Cardiac Surgery	1.026	[0.740–1.422]	*0.8768*	0.939	[0.726–1.214]	*0.6304*
**Donor**						
**Age**	1.007	[0.991–1.023]	*0.4103*	1.000	[0.987–1.012]	*0.9605*
**Sex**						
Male	REF			REF		
Female	0.996	[0.657–1.509]	*0.9838*	0.943	[0.673–1.320]	*0.7310*
**Body Mass Index**	0.982	[0.957–1.006]	*0.1451*	0.991	[0.971–1.011]	*0.3646*
**Race**						
Non-Hispanic/Latino White						
Non-Hispanic/Latino Black	1.518	[0.915–2.519]	*0.1064*	1.194	[0.824–1.730]	*0.3479*
Hispanic/Latino	1.441	[0.965–2.151]	*0.0738*	1.036	[0.758–1.416]	*0.8249*
Non-Hispanic/Latino Asian	1.132	[0.398–3.223]	*0.8160*	0.865	[0.352–2.127]	*0.7517*
Non-Hispanic/Latino Other	1.409	[0.296–6.702]	*0.6667*	1.228	[0.547–2.758]	*0.6187*
**Left Ventricular Ejection Fraction**	1.010	[0.986–1.034]	*0.4060*	0.999	[0.981–1.017]	*0.9044*
**Comorbidities**						
Diabetes	0.991	[0.521–1.885]	*0.9774*	1.078	[0.549–2.117]	*0.8281*
Smoking	0.788	[0.460–1.350]	*0.3852*	1.139	[0.752–1.726]	*0.5396*
Cocaine	0.876	[0.589–1.303]	*0.5139*	0.925	[0.699–1.224]	*0.5856*
Alcohol abuse	0.922	[0.605–1.407]	*0.7073*	0.863	[0.629–1.185]	*0.3621*
Hypertension	1.175	[0.779–1.774]	*0.4413*	1.153	[0.821–1.617]	*0.4117*
Intravenous drug use	1.235	[0.772–1.975]	*0.3793*	0.880	[0.619–1.250]	*0.4742*
**Donor Type**						
Donation after Brain Death	REF			REF		
Donation after Cardiac Death	0.409	[0.193–0.870]	***0.0202***	1.101	[0.561–2.163]	*0.7791*
**Donor Perfusion**	4.053	[1.618–10.156]	***0.0028***	0.504	[0.241–1.054]	*0.0686*

**Table 4:** Outcomes stratified by donor type.

Variables	Early PPM				Late PPM			
	Total	DBD (N = 329)	DCD (N = 25)	*p Value*	Total	DBD (N = 329)	DCD (N = 25)	*p Value*
**Death**								
Death overall, N (%)	56	54 (16.4%)	2 (8%)	*0.2664*	78	78 (14.5%)	0 (0%)	*0.1004*
Death within 30 days, N (%)	7	6 (1.8%)	1 (4%)	*0.4512*	2	2 (0.4%)	0 (0%)	*0.8070*
Death within 1 year, N (%)	28	26 (7.9%)	2 (8%)	*0.9861*	28	28 (5.2%)	0 (0%)	*0.3490*
Death within 5 years, N (%)	54	52 (15.8%)	2 (8%)	*0.2954*	73	73 (13.6%)	0 (0%)	*0.1138*
**Graft Failure**								
Graft Failure overall, N (%)	18	17 (5.2%)	1 (4%)	*0.7979*	22	22 (4.1%)	0 (0%)	*0.4091*
Graft Failure within 30 days, N (%)	7	6 (1.8%)	1 (4%)	*0.4512*	1	1 (0.2%)	0 (0%)	*0.8630*
Graft Failure within 1 year, N (%)	14	13 (4%)	1 (4%)	*0.9904*	8	8 (1.5%)	0 (0%)	*0.6232*
Graft Failure within 5 years, N (%)	18	17 (5.2%)	1 (4%)	*0.7979*	22	22 (4.1%)	0 (0%)	*0.4091*
**Primary Outcome**								
Primary Graft Dysfunction, N (%)	15	9 (2.7%)	6 (24%)	***<0.0001***	3	3 (0.6%)	0 (0%)	*0.7646*
Left Primary Graft Dysfunction, N (%)	9	5 (1.5%)	4 (16%)	***<0.0001***	3	3 (0.6%)	0 (0%)	*0.7646*
Right Primary Graft Dysfunction, N (%)	10	5 (1.5%)	5 (20%)	***<0.0001***	3	3 (0.6%)	0 (0%)	*0.7646*
**Secondary Outcome**								
Length of Hospital Stay, MEAN (STD)	32.04 (27.18)	31.88 (26.73)	34 (32.92)	*0.7083*	27.33 (23.55)	27.32 (23.71)	27.56 (18.01)	*0.9683*
Stroke, N (%)	17	16 (4.9%)	1 (4%)	*0.8457*	15	15 (2.8%)	0 (0%)	*0.4983*
Dialysis, N (%)	87	76 (23.1%)	11 (44%)	***0.0193***	107	103 (19.1%)	4 (25%)	*0.5588*
Acute Rejection, N (%)	41	37 (11.3%)	4 (16%)	*0.4740*	40	39 (7.3%)	1 (6.3%)	*0.8791*
Treated for Rejection within 1 year, N (%)	47	46 (14%)	1 (4%)	*0.1562*	80	78 (14.5%)	2 (12.5%)	*0.8227*
Reintubation, N (%)	12	11 (3.3%)	1 (4%)	*0.8612*	3	3 (0.6%)	0 (0%)	*0.7646*
Airway Dehiscence, N (%)	3	3 (0.9%)	0 (0%)	*0.6316*	2	2 (0.4%)	0 (0%)	*0.8070*

**Table 5:** Multivariate logistic regression for mortality following PPM implantation.

Variable	Early PPM			Late PPM		
	aHR	[95% CI]	*p Value*	aHR	[95% CI]	*p Value*
**Permanent Pacemaker prior to Discharge**						
No	REF			REF		
Yes	1.162	[0.760–1.776]	*0.4879*	0.748	[0.542–1.032]	*0.0767*
**Recipient**						
**Age**	1.009	[0.992–1.027]	*0.3125*	1.015	[1.000–1.030]	***0.0429***
**Sex**						
Male	REF			REF		
Female	1.659	[0.917–3.001]	*0.0944*	1.374	[0.893–2.113]	*0.1480*
**Body Mass Index**	1.013	[0.987–1.040]	*0.3316*	1.000	[1.000–1.000]	***0.0007***
**Race**						
Non-Hispanic/Latino White	REF			REF		
Non-Hispanic/Latino Black	1.113	[0.666–1.859]	*0.6836*	1.439	[0.999–2.074]	*0.0509*
Hispanic/Latino	1.476	[0.765–2.849]	*0.2453*	1.214	[0.706–2.086]	*0.4835*
Non-Hispanic/Latino Asian	1.366	[0.428–4.361]	*0.5981*	1.270	[0.574–2.811]	*0.5550*
Non-Hispanic/Latino Other	1.750	[0.433–7.068]	*0.4319*	1.397	[0.297–6.572]	*0.6718*
**Distance from Donor Hospital to Transplant Center**	0.999	[0.998–0.999]	***0.0237***	1.000	[0.999–1.001]	*0.4921*
**Ischemic Time in Hours**	1.258	[1.089–1.453]	***0.0018***	1.187	[1.009–1.398]	***0.0389***
**Preop Hemodynamics**						
Mean Pulmonary Artery Pressure	0.626	[0.089–4.381]	*0.6367*	0.883	[0.194–4.021]	*0.8722*
Pulmonary Capillary Wedge Pressure	1.162	[0.432–3.126]	*0.7662*	0.647	[0.300–1.393]	*0.2653*
Cardiac Output	1.462	[0.16–13.322]	*0.7364*	2.064	[0.343–6.408]	*0.4284*
**Cardiac Support Devices at Transplant**						
Extra-Corporeal Membrane Oxygenation	1.037	[0.379–2.837]	*0.9433*	1.416	[0.703–2.850]	*0.3301*
Ventilator	1.018	[0.27–3.837]	*0.9788*	1.700	[0.672–4.305]	*0.2628*
**Comorbidities**						
Steroid	0.813	[0.410–1.613]	*0.5541*	0.917	[0.475–1.771]	*0.7971*
Smoking	1.353	[0.850–2.153]	*0.2027*	1.048	[0.750–1.463]	*0.7836*
Malignancy	1.039	[0.397–2.715]	*0.9384*	1.469	[0.898–2.404]	*0.1259*
Prior Cardiac Surgery	1.476	[0.951–2.293]	*0.0828*	1.397	[1.003–1.947]	***0.0480***
**Donor**						
**Age**	1.018	[0.997–1.039]	*0.0987*	1.008	[0.991–1.025]	*0.3555*
**Sex**						
Male	REF			REF		
Female	0.746	[0.412–1.351]	*0.3332*	0.823	[0.534–1.268]	*0.3768*
**Body Mass Index**	0.979	[0.946–1.014]	*0.2387*	1.003	[0.979–1.027]	*0.8284*
**Race**						
Non-Hispanic/Latino White						
Non-Hispanic/Latino Black	1.398	[0.728–2.686]	*0.3144*	1.268	[0.798–2.015]	*0.3141*
Hispanic/Latino	1.133	[0.660–1.944]	*0.6498*	0.928	[0.598–1.439]	*0.7375*
Non-Hispanic/Latino Asian	0.814	[0.170–3.898]	*0.7971*	0.971	[0.298–3.161]	*0.9612*
Non-Hispanic/Latino Other	-	-	-	0.778	[0.232–2.607]	*0.6841*
**Left Ventricular Ejection Fraction**	1.014	[0.987–1.043]	*0.3063*	1.008	[0.983–1.034]	*0.5106*
**Comorbidities**						
Diabetes	2.379	[1.212–4.671]	***0.0118***	1.222	[0.544–2.746]	*0.6280*
Smoking	0.641	[0.267–1.538]	*0.3188*	1.159	[0.678–1.981]	*0.5890*
Cocaine	0.890	[0.533–1.487]	*0.6569*	1.303	[0.920–1.845]	*0.1355*
Alcohol abuse	0.861	[0.476–1.558]	*0.6216*	0.788	[0.515–1.208]	*0.2745*
Hypertension	0.572	[0.317–1.032]	*0.0637*	1.192	[0.791–1.798]	*0.4009*
Intravenous drug use	0.918	[0.473–1.782]	*0.8001*	0.939	[0.595–1.482]	*0.7866*
**Donor Type**						
Donation after Brain Death	REF			REF		
Donation after Cardiac Death	0.309	[0.093–1.027]	*0.0553*	0.673	[0.214–2.118]	*0.4986*
**Donor Perfusion**	1.853	[0.553–6.217]	*0.3176*	1.203	[0.366–3.951]	*0.7605*

## Data Availability

Data is available upon request from UNOS/OPTN.

## Supplementary Materials

The following supporting information can be downloaded at: https://www.mdpi.com/article/10.3390/jcm15134895/s1, Figure S1: Patient selection; Figure S2: Matched cohort for early pacemakers, Figure S3: Matched cohort for late pacemakers.

## Abbreviations

The following abbreviations are used in this manuscript:

AV	Atrioventricular
CI	Confidence interval
DBD	Donation after brain death
DCD	Donation after circulatory death
HR	Hazard ratio
HT	Heart transplantation
aOR	Adjusted odds ratio
PGD	Primary graft dysfunction
PPM	Permanent pacemaker
PSM	Propensity score matching
SND	Sinus node dysfunction
UNOS	United Network for Organ Sharing
