# Supplementary figures and images for: Heart Transplantation Requiring Permanent Pacemaker: Risk Factors and Outcomes
Source: J Clin Med. 2026 Jun 24;15(13):4895. doi: 10.3390/jcm15134895 (PMC13361714; doi:10.3390/jcm15134895)

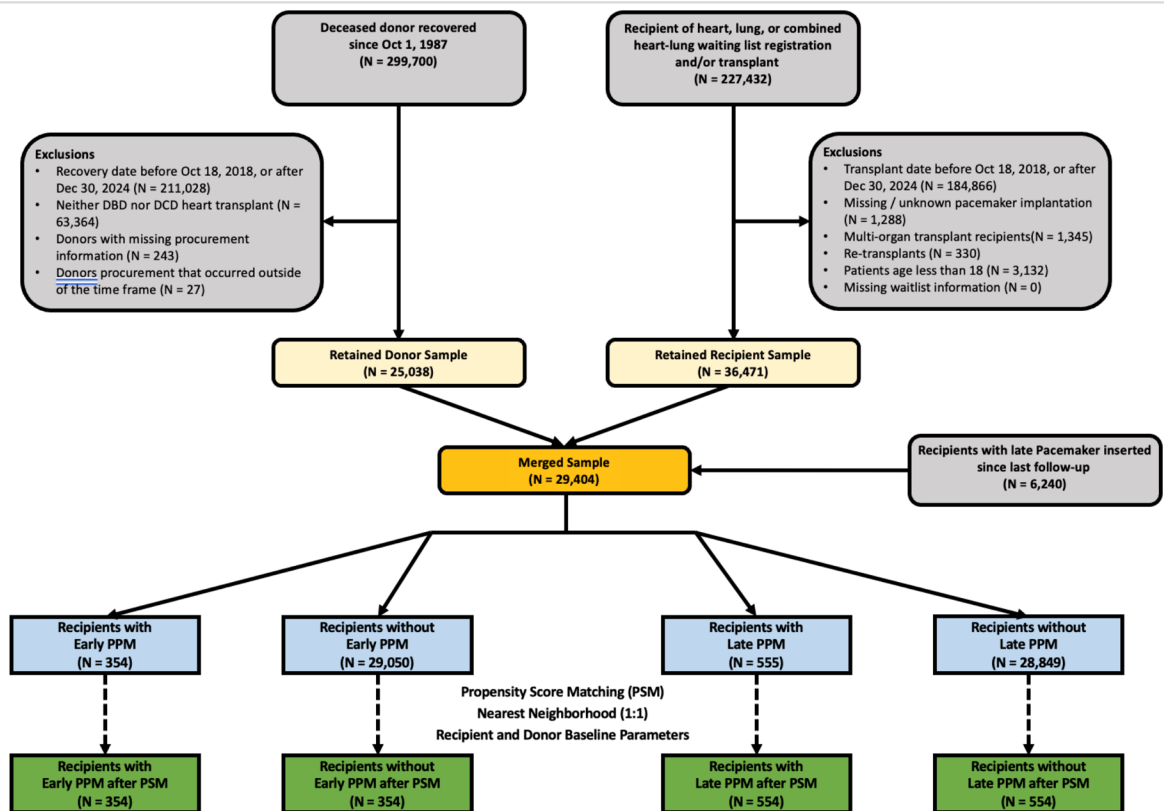

Supplement: Supplementary file 1 [file jcm-15-04895-s001.zip › Figure S1. Patient selection.pdf]

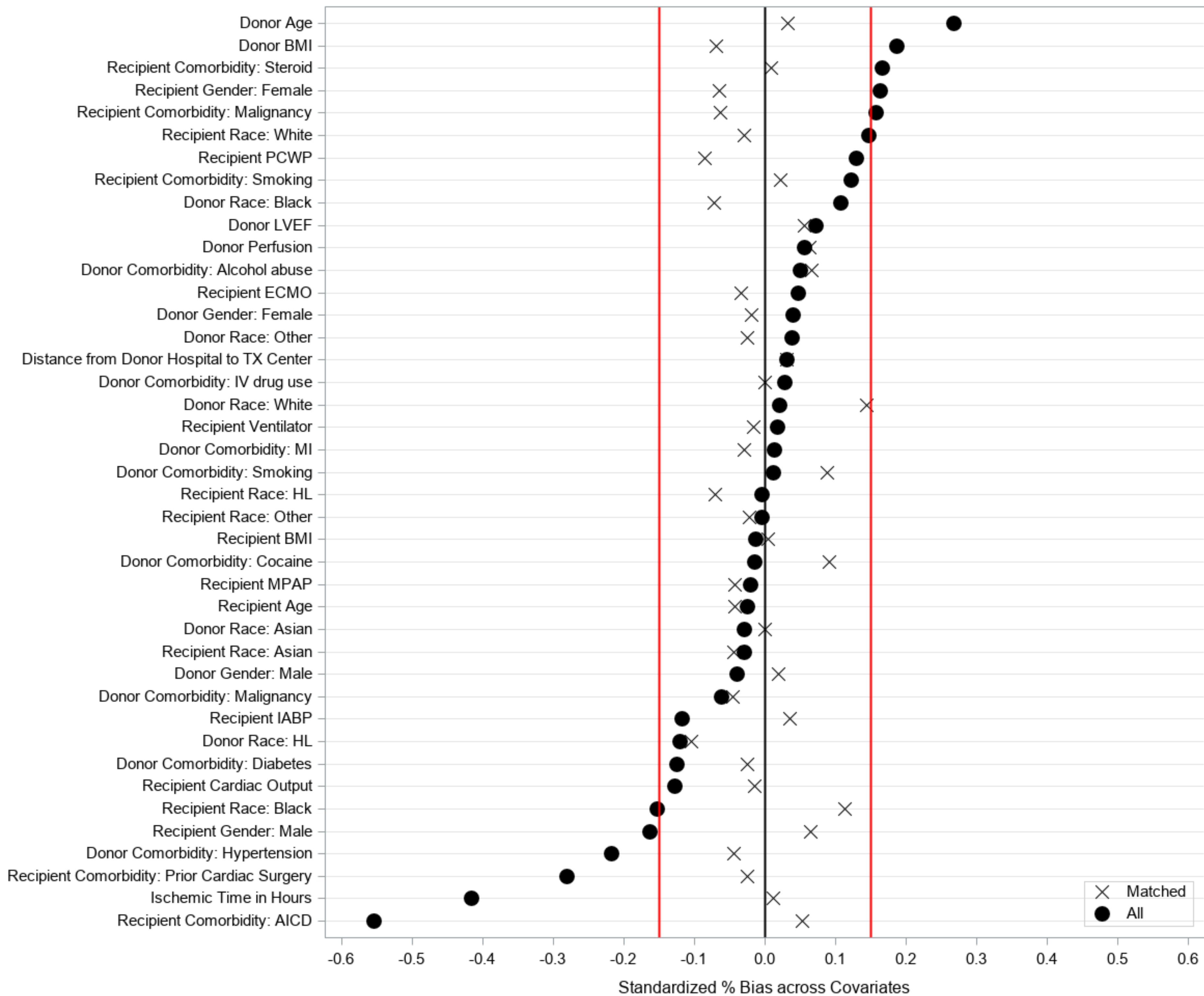

Supplement: Supplementary file 1 [file jcm-15-04895-s001.zip › Figure S2. Matched cohort for early pacemakers.pdf]

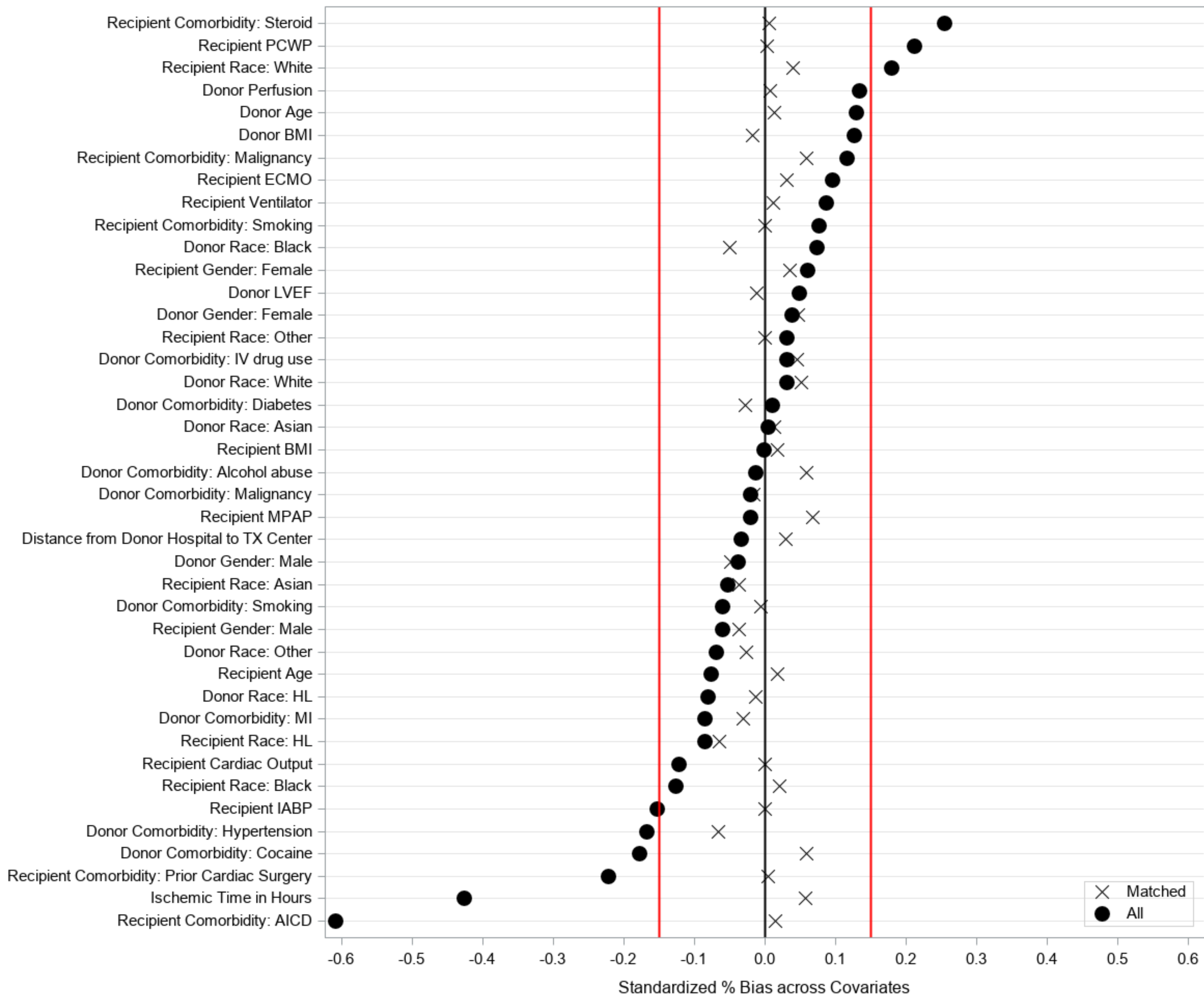

Supplement: Supplementary file 1 [file jcm-15-04895-s001.zip › Figure S3. Matched cohort for late pacemakers.pdf]
